# Uncovering a hidden diversity: optimized protocols for the extraction of dsDNA bacteriophages from soil

**DOI:** 10.1186/s40168-020-0795-2

**Published:** 2020-02-11

**Authors:** Pauline C. Göller, Jose M. Haro-Moreno, Francisco Rodriguez-Valera, Martin J. Loessner, Elena Gómez-Sanz

**Affiliations:** 1 Institute of Food, Nutrition and Health, ETH Zurich, 8092 Zurich, Switzerland; 2 Departamento de Producción Vegetal y Microbiología, Universidad Miguel Hernández, San Juan de Alicante, Alicante, Spain; 3 Moscow Institute of Physics and Technology, Dolgoprudny, 141701 Russia; 4 Área de Microbiología Molecular, Centro de Investigación Biomédica de La Rioja (CIBIR), Logroño, Spain

**Keywords:** Bacteriophage extraction, Phage virome, Soil samples, Bacterial contamination, Spike-in

## Abstract

**Background:**

Bacteriophages (phages) are the most numerous biological entities on Earth and play a crucial role in shaping microbial communities. Investigating the bacteriophage community from soil will shed light not only on the yet largely unknown phage diversity, but may also result in novel insights towards their functioning in the global biogeochemical nutrient cycle and their significance in earthbound ecosystems. Unfortunately, information about soil viromes is rather scarce compared to aquatic environments, due to the heterogeneous soil matrix, which rises major technical difficulties in the extraction process. Resolving these technical challenges and establishing a standardized extraction protocol is, therefore, a fundamental prerequisite for replicable results and comparative virome studies.

**Results:**

We here report the optimization of protocols for the extraction of phage DNA from agricultural soil preceding metagenomic analysis such that the protocol can equally be harnessed for phage isolation. As an optimization strategy, soil samples were spiked with *Listeria* phage A511 (Myovirus), *Staphylococcus* phage 2638AΔLCR (Siphovirus) and *Escherichia* phage T7 (Podovirus) (each 10^6^ PFU/g soil). The efficacy of phage (i) elution, (ii) filtration, (iii) concentration and (iv) DNA extraction methods was tested. Successful extraction routes were selected based on spiked phage recovery and low bacterial 16S rRNA gene contaminants. Natural agricultural soil viromes were then extracted with the optimized methods and shotgun sequenced. Our approach yielded sufficient amounts of inhibitor-free viral DNA for shotgun sequencing devoid of amplification prior library preparation, and low 16S rRNA gene contamination levels (≤ 0.2‰). Compared to previously published protocols, the number of bacterial read contamination was decreased by 65%. In addition, 379 novel putative complete soil phage genomes (≤ 235 kb) were obtained from over 13,000 manually identified viral contigs, promising the discovery of a large, previously inaccessible viral diversity.

**Conclusion:**

We have shown a considerably enhanced extraction of the soil phage community by protocol optimization that has proven robust in both culture-dependent as well as through viromic analyses. Our huge data set of manually curated soil viral contigs substantially increases the amount of currently available soil virome data, and provides insights into the yet largely undescribed soil viral sequence space.

## Introduction

Soil bacteriophages are a vital part of bacterial ecology, as they shape soil microbial communities through facilitating horizontal gene transfer, and constitute a major reservoir of genetic material that contributes to biological evolution and diversity [1–4]. Soil is known to harbour a vast abundance of phages (10^7^–10^9^ gdw^−1^), with their numbers exceeding those of co-occurring bacteria by 10 to 1000-fold [5–7]. This undiscovered viral diversity could lead not only to novel findings into phage biology, but its characterization may also result in exciting insights towards their functioning in the global biogeochemical nutrient cycle and their significance in terrestrial ecosystems. Despite this ecological importance, the soil virome is particularly poorly studied. This circumstance is emphasized by the limited data on publicly available soil viromes, which represent only 1.8% of all accessible viromes, whereas controversially, 97% of all viruses are thought to be found in solid matrixes as soil and sediment [8]. This narrow exploration likely results from major technical difficulties in phage isolation, which arise due to the utter microheterogeneity of soils, the presence of organic inhibitors that interfere with many molecular biology techniques and a lack of appropriate screening tools. Given the explained physicochemical diversity of soil, its matrix complexity and high microbial diversity [2, 9], it is not surprising that no universal phage extraction protocol or standardization towards viral elution, concentration and DNA extraction have yet been proposed. Some phage extraction protocols for soil samples have been suggested in the literature [1, 5, 10–21], of which only few have experimentally optimized the process of viral recovery [1, 5, 17, 21]. Those phage extraction protocols need consistency to compare changes in viral abundance within and across soil samples, and require DNA amplification prior to library preparation for metagenomic analysis [13, 16, 20, 22]. Moreover, the routinely applied (rather harsh) methods may render phage isolation impossible [19]. Among them, soil phage extraction has included a wide range of elution media, such as deionized water [14, 22], saline magnesium (SM) buffer [11, 17, 23], potassium citrate buffer [6], 10% beef extract [5, 24], amended potassium citrate buffer (AKC) [1], Na/K buffer [12] or phosphate-buffered saline (PBS) supplemented with beef extract [18] (some reviewed in [2, 17]). Those elution media are commonly combined with mechanical approaches to disrupt phage soil interactions, such as homogenization [18], sonication [5, 6, 25], vortexing [1, 5, 17], shaking [11, 16, 22], magnetic stirring [24], blending [12, 26] or bead-beating [10, 26]. Despite these diverse approaches, the elution and recovery of soil phages remains the major bottleneck in the extraction, considering the high absorption of viruses to soil particles (> 90%) [2, 27]. Soil viruses are therefore unintentionally removed by centrifugation or filtration techniques in the very first steps of most protocols. For soil phage concentration, a universal approach is likely to be found, which could include typical techniques as tangential flow filtration (TFF) [23], or polyethylene glycol concentration (PEG) [11, 23]. Those concentration techniques are occasionally combined with caesium chloride (CsCl) ultracentrifugation for purification, but have unfortunately only been evaluated for efficiency in other sample matrixes [28], or resulted in uncertain conclusions [21].

For functional and sequencing viromics, purified phage DNA without contaminating bacterial or eukaryotic sequences is critical for experimental success. However, the selective extraction of viral DNA from any source, including soil matrixes, has shown to be challenging, since most extracted phage viromes show bacterial DNA contamination above the proposed limit for a pure virome (> 0.2‰ of ribosomal DNA reads) [22, 29]. No studies have yet assessed the influence of different phage elution, concentration and DNA extraction methods concerning soil bacteriophage diversity and, importantly, bacterial contaminants, using plaque assay and metagenomics. Here, we report the optimization of protocols for the extraction of dsDNA bacteriophages from soil samples that can be used prior to metagenomics and equally be applied to infective phage particle isolation from soil. The advantage of using a culture-dependent detection method for viral recovery is evident when considering phage isolation and the obvious viability of phages used for DNA extraction. For this, soil samples were spiked with a viral community consisting of phages from different families of *Caudovirales*, and the efficiency of several bacteriophage elution, concentration and DNA extraction procedures were determined. Successful extraction routes were selected based on spiked phage recovery and low bacterial 16S rRNA gene contaminants. Natural agricultural soil viromes were then extracted with the optimized methods and shotgun sequenced. Our approach yielded sufficient amounts of inhibitor-free viral DNA for shotgun sequencing without any amplification step prior to library preparation, and low 16S rRNA gene contamination levels (≤ 0.2‰). Compared to previously published protocols, the number of bacterial read contamination could be decreased by 65%. In total, we obtained 379 novel, putative circularized soil phage genomes of up to 235 kb in size, from > 13,000 manually curated viral contigs. This data set greatly extends the amount of today’s available soil viral contigs, and opens the door for the discovery of a remarkably diverse soil virome.

## Results

### Protocol optimization strategy

The selective criteria for optimized extraction routes relied on three major parameters: (i) phage yield at each step of the extraction route, (ii) reduction of bacterial DNA contamination levels and (iii) bias minimization in the relative abundance and diversity of viruses. The efficacy of different bacteriophage elution, concentration and DNA extraction procedures was determined by monitoring spiked phage recovery by plaque assay and bacterial contamination levels by qPCR (Additional file 1: Figure S1). For this, a mock viral community consisting of phages from different families: *Listeria* phage A511 (Myovirus), *Staphylococcus* phage 2638AΔLCR (Siphovirus) and *Escherichia* phage T7 (Podovirus), was spiked (each phage at 10^6^ PFU/g soil) into agricultural soil samples. Successful extraction routes were then shotgun sequenced to gain a deeper understanding of the soil viral community and to compare the soil viral diversity in each extraction route, including data generated from a literature-based approach.

### Resuspension of soil phages

**Fig. 1:**
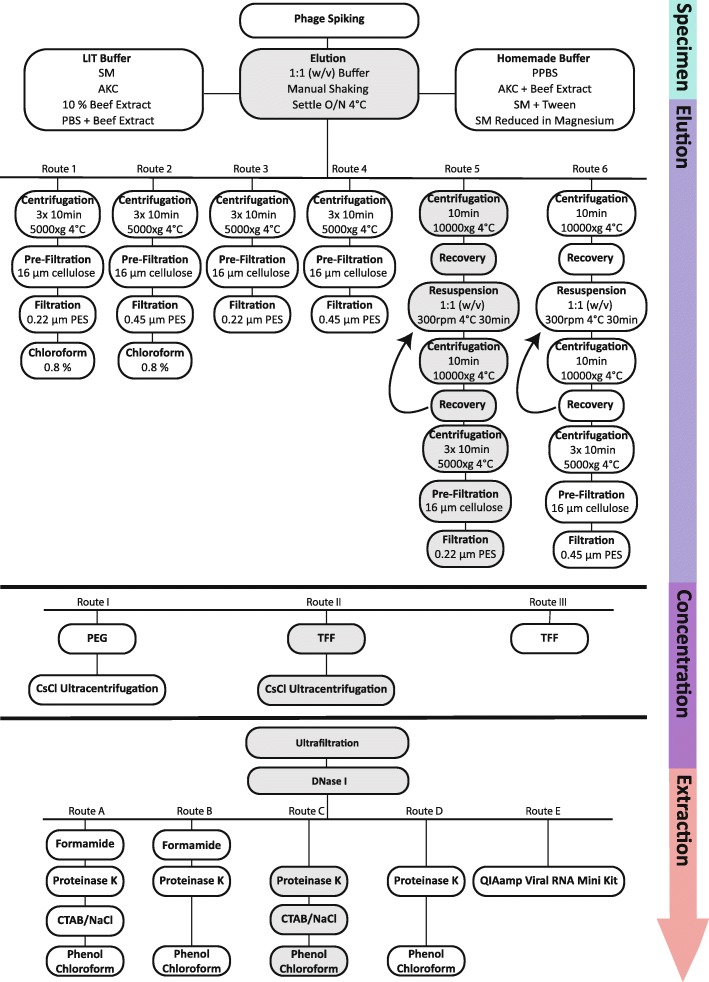
Optimization protocols for bacteriophage elution and extraction. Optimization strategy for phage DNA extraction from soil samples prior metagenomic analysis. An efficient concentration and purification of soil bacteriophages together with a complete removal of bacterial DNA is anticipated. Bacteriophage elution (route 1–6), concentration (route I–III) and DNA extraction (route A–E) methods are evaluated for efficiency using a spiked soil sample (A511, 2638AΔLCR, T7 at 10^6^ PFU/g soil) and plaque assays. Bacterial DNA depletion was assessed using 16S rRNA qPCR. Optimal phage extraction route is highlighted in grey as verified by sequencing metagenomics. *LIT* literature suggested buffer, *SM* saline magnesium, *AKC* amended potassium citrate, *PBS* phosphate**-**buffered saline, *O*/*N* overnight, *PPBS* protein supplemented PBS, *w*/*v* weight to volume, *PES* polyether sulfone, *TFF* tangential flow filtration, *CsCl* caesium chloride, *CTAB* cetyltrimethylammonium bromide, *NaCl* sodium chloride.

**Fig. 2:**
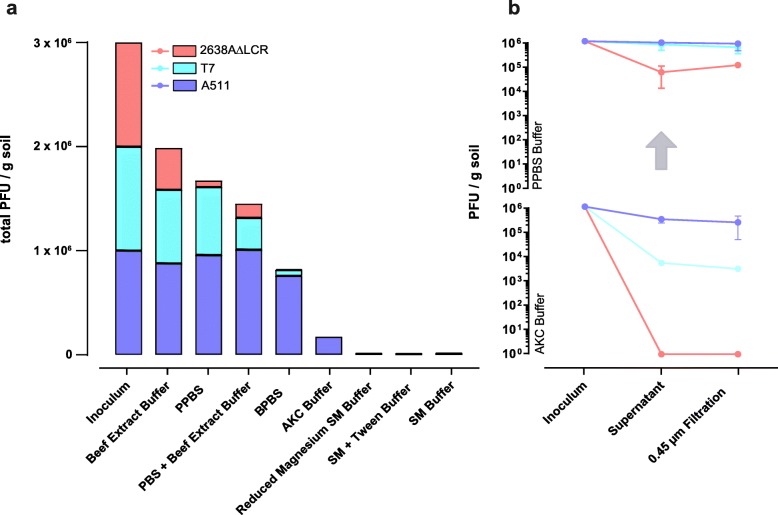
Bacteriophage elution buffers for soil samples. Bacteriophage elution from soil samples was assessed for different elution buffers. The ability to elute phages from soil samples was tested for elution buffers previously published in literature or novel constituted elution buffer. Soil samples were spiked with an artificial viral community (A511, 2638AΔLCR, T7 at 10^6^ PFU/g soil) and bacteriophage yield was assessed using plaque assay. (**a**) SM buffer [11, 23]; 10% beef extract buffer [5, 24]; PBS supplemented with beef extract [18]; AKC [1]. (**b**) Elution amelioration measured by plaque assay (mean ± SD) when using optimized PPBS instead of literature proposed elution buffer.

A proper suspension of bacteriophages from soil particles is crucial to retain viral diversity and reproducibility. We compared the most promising elution buffers found in the literature to maximise bacteriophage suspension from agricultural soil samples (Fig. 1). For this, the most commonly used (or elsewhere optimized) elution buffers such as SM buffer [11, 23], AKC [1], 10% beef extract [5, 24] or PBS amended with beef extract [18] were assessed and compared in viral yield using plaque assay. When soil samples were suspended using SM or AKC buffer, as little as 0.5 to 5% of all spiked phages were recovered (Fig. 2a, b). Those simple salt-supplemented buffers, however, provided good filtration properties after soil suspension and did not interfere with any downstream analysis. Protein-supplemented elution buffers, such as PBS + 2.5% beef extract or 10% beef extract, recovered a compelling number of spiked bacteriophages (51.1% and 66.7%, respectively). However, when performing phage elution protocols with more than 300 g of soil, any buffer that contained beef extract resulted in being a poor choice. Vacuum-filtration attempts with a filter pore size smaller than 1 μm were instantly clogged and further techniques applied downstream (qPCR, microscopy or concentration methods) failed completely. It is therefore evident that beef extract is a very efficient supplement to suspend soil phages, but equally dissolves other organic compounds that interfere with ensuing techniques.

Our elution buffers were, therefore, adapted and assessed for efficiency with the following modifications: SM buffer was supplemented with 0.01% tween or reduced in magnesium, and additionally, two novel in-house elution buffers were designed consisting of PBS with either a bovine serum albumin (BSA) (PPBS) or beef extract (BPBS) supplementation. For PPBS (protein supplemented PBS), the 10% PBS, 1% potassium citrate and 150 mM MgSO_4_ were adapted from the optimized AKC buffer [1], while ethylenediaminetetraacetic acid (EDTA) was removed and substituted with a 2% BSA supplementation (Fig. 1). For beef extract supplemented PBS (BPBS), BSA from PPBS was replaced by beef extract. Salt components in these elution buffers serve as pH and viral particle stabiliser [1], whereas the protein addition as BSA or beef extract offers viral binding sites and disrupts soil-viral interactions [24]. The adjusted SM buffer failed to improve recovery and, hence, did not prevent non-specific phage soil interactions. PPBS performed only negligibly worse in the elution of bacteriophages compared to 10% beef extract (55.8% vs. 66.2% recovery, respectively), and did not trigger any of the technical difficulties described above, even when applied to large samples (> 1 kg soil). Interestingly, when replacing BSA with beef extract (BPBS) in this optimized buffer, no enhanced phage recovery was observed. As previously suggested [6, 12], recovery of spiked bacteriophages was further optimized by resuspending the soil pellet thrice (Fig. 1, route 5–6), resulting in a relative increase in recovery of 48% compared to a single suspension step (45.7% and 67.3% recovery, respectively). Our optimized elution protocol allows, hence, a virtually complete recovery of infective bacteriophages with simple methods such as adjusting elution buffer constitution and washing rounds of the soil pellet (Fig. 2b). In summary, soil samples should be eluted in equal volumes of PPBS, manually shaken by inversion and left to settle overnight at 4 °C. The suspended soil should then be centrifuged as described in Fig. 1, the supernatant kept aside and the pellet resuspended in another volume of PPBS for a total of three rounds [6, 12].

### Removal of bacterial contaminants using centrifugation and filtration

**Fig. 3:**
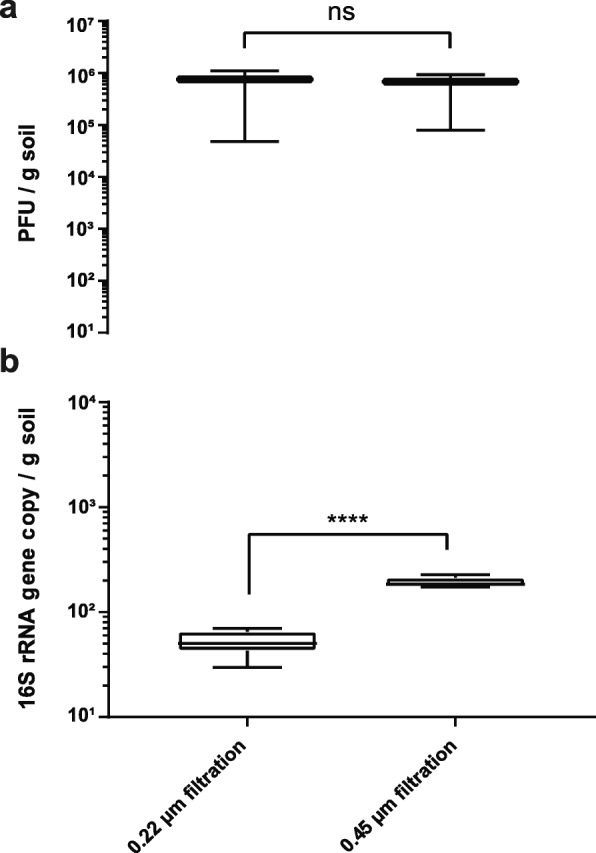
Effect of filtration pore sizes on bacteriophage recovery and bacterial contamination. Recovery of spiked bacteriophages (**a**) and respective bacterial DNA contamination (**b**) with a 0.22 μm or 0.45 μm PES filtration. Spiked bacteriophages were detected after bacteriophage elution and filtration of the soil suspension using plaque assay. Bacterial DNA contamination was assessed using Taqman qPCR amplifying the 16S rRNA gene. Boxplot and whiskers (min to max) with ns equals *p* = 0.7782 (*n* = 9), **** equals *p* < 0.0001 (*n* = 6).

After elution optimization of bacteriophages from soil particles, the complete removal of contaminating bacterial cells was attempted. In literature, several techniques such as centrifugation steps prior a 0.8 μm [30], 0.45 μm [1, 26, 31] or 0.22 μm [5, 11–13, 16, 23, 32, 33] polyethersulfone (PES) filtration (Fig. 1, route 3–6), sometimes coupled with a chloroform treatment [12, 23] (Fig. 1, route 1–2), are suggested. PES membranes have previously resulted in a higher percentage of viral reads and improved reduction of bacterial contaminants, when compared to polycarbonate (PC) or centrifugal (PVDF) membranes, and should thus be applied consistently [30]. The utilization of such diverse filter pore size in literature has likely arisen by the fear of a compromising effect on the viral community through smaller filter pores. A reductive effect on soil phage diversity when using a 0.22 μm filter has however not been demonstrated yet. In this optimization protocol, any filtration attempt with pore size < 16 μm was impaired if the single (Fig. 1, route 1–4) or united (Fig. 1, route 5–6) supernatants were not centrifuged thrice at 5000×*g* for 10 min beforehand. Filtration procedures and the spiked phage community though were measurably not impaired when using low-speed centrifugation to remove impurities. No significant difference in spiked phage recovery was observed when comparing a 0.22 μm to 0.45 μm filter pore size (unpaired *t* test, *p* value = 0.7782, *n* = 9) (Fig. 3a). In addition, a 16S rRNA qPCR analysis revealed that both the 0.45 μm and 0.22 μm PES filtration techniques removed > 99.9% of all bacterial 16S rRNA genes, whereat the latter decreased bacterial contamination significantly better (unpaired *t* test, *p* value < 0.0001, *n* = 6) (Fig. 3b). Yet, a higher recovery of phages in viromic samples was recently suggested when substituting a 0.22 μm for a 0.45 μm filtration step [31]. Along these lines, the 0.45 μm filtration was nevertheless chosen here for further optimization purposes in order to avoid a potential bias in the native soil viral community and both routes (0.22 μm and 0.45 μm filtration) were selected for shotgun sequencing analysis (see below).

Besides centrifugation and filtration, the efficiency of chloroform treatment to remove bacterial contamination was assessed (Fig. 1, route 1–2). Chloroform treatment is generally a rather impractical approach, because both bacteriophages in the environmental sample [23], as well as downstream concentration devices, are sensitive to chloroform. A maximum concentration of 0.8% chloroform is supported when using a PES tangential—or regular filtration, which in turn did not reduce bacterial DNA contamination (data not shown). A chloroform treatment prior to phage concentration was therefore excluded from the protocol.

### Concentration of viral particles from soil samples prior to DNA extraction

**Fig. 4:**
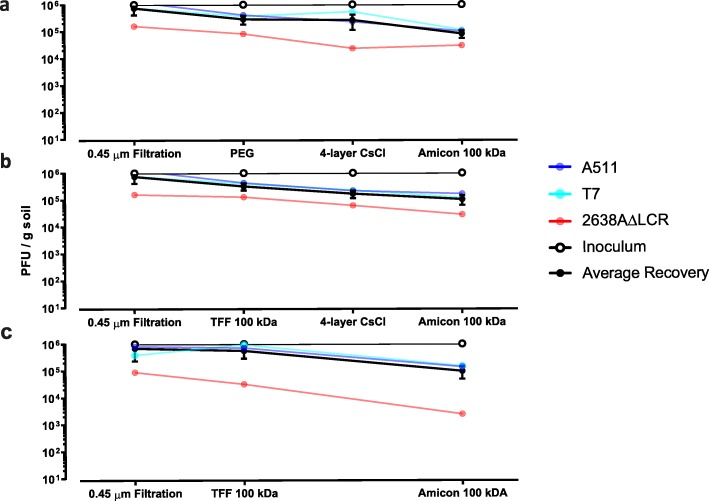
Recovery of spiked bacteriophages. Soil samples were spiked with an artificial viral community (phage A511, 2638AΔLCR, T7 at 10^6^ PFU/g soil), and phages were eluted with the optimized protocol. Spiked bacteriophage recovery was then monitored in concentration and purification methods. A reduction in PFU with a given technique quantifies (mean ± SEM in triplicates) the loss of spiked bacteriophages. (**a**) Spiked phage recovery with PEG concentration and CsCl purification. (**b**) Spiked phage recovery with TFF and CsCl purification. (**c**) Spiked phage recovery with TFF without further purification.

PEG and TFF concentration techniques, in combination with ultrafiltration, are commonly used techniques to concentrate viral particles from large volumes. These techniques have been described in detail elsewhere [11, 23, 28, 34] and were assessed for functionality in eluted soil samples. An optimal concentration technique should concentrate viral particles without introducing a bias to the native viral community, and equally reduce the suspensions’ volume sufficiently to allow DNA extraction. Spiked phages were eluted from soil samples using the optimized elution protocol, filtrated through a 0.45 μm PES filter and subjected to PEG or TFF (Fig. 1, route I–II). As shown in Fig. 4, both concentration techniques performed equally well in concentrating spiked phages and no differences in spiked phage yield after concentration was observed. Furthermore, viral suspensions were reduced in both approaches to a final volume of 20–50 ml, which allowed CsCl ultracentrifugation and ultrafiltration. Similar to filtration, the spike-dependent approach for evaluating the efficiency of those concentration methods has not resulted in a clear conclusion. Both routes were therefore selected for shotgun sequencing to elucidate the superior technique for viral recovery and diversity (see below). CsCl ultracentrifugation concentrates and purifies phages from environmental samples or pure cultures. It is, however, not only technically demanding but also cost and equipment intensive. The necessity of a CsCl purification for soil samples prior to DNA extraction was evaluated by extracting soil viruses with and without CsCl centrifugation prior to ultrafiltration (Fig. 1, route II and III). Ultrafiltration units were coated with PBS + 1% BSA to reduce viral absorption as suggested and optimized elsewhere [34]. Purification of soil samples with CsCl ultracentrifugation seemed to slightly diminish spiked viral yields (Fig. 4a, b). However, this loss in PFU could also be attributed to a loss in infectivity rather than a loss in viral yield caused by centrifugation conditions [28]. When avoiding a CsCl purification step prior to ultrafiltration (Fig. 1, route III, and Fig. 4c), the centrifugal filters clogged while concentrating and the remaining volume could not be reduced below 5 ml. A purification of soil viral suspensions using CsCl ultracentrifugation was therefore implemented in the optimized protocol, which ensured a concentration to a final volume below 300 μl. When aiming for isolating infective viral particles from soil samples, however, this purification can easily be omitted.

### Phage DNA extraction from soil samples

Ultrafiltrated concentrated samples were treated with DNase I to remove free DNA that is not enclosed by viral capsids and brought up to a final volume of 600 μl. From here, phage DNA was either extracted using modified phenol/chloroform extraction routes (Fig. 1, route A–D) or QIAamp Viral RNA Mini Kit (QIAGEN) according to manufacturer instructions (Fig. 1, Route E). DNA extraction using the suggested kit resumed in a > 10-fold reduction of viral DNA than other proposed DNA extraction routes, and was therefore excluded from the optimized protocol (data not shown). The phenol/chloroform DNA extraction was adapted from Thurber et al. [23], whereas the necessity of formamide and cetyltrimethylammonium bromide (CTAB) in both viral DNA extraction routes was assessed by bypassing these steps singularly or in combination. DNA extraction routes with larger volumes that originated directly from the TFF concentrated sample (Fig. 1, route III, no CsCl purification) resulted in a jellification of the sample after the addition of formamide, or presented contamination with qPCR inhibitors that even flawed DNA measurements if formamide was neglected. Extraction routes covering a formamide treatment resulted therefore either in a complete loss of viral DNA through a jellification of the sample (Fig. 1, route III), or reduction in DNA yield from CsCl purified routes (Additional file 3: Table S2). Formamide treatment decreased the viral DNA yield irrespectively of purification and was therefore excluded. On the other hand, a CTAB/NaCl treatment did not correlate consistently with a disadvantageous outcome and was further highly dependent on the experimental setup. For the sake of simplicity, a CTAB/NaCl treatment of viral suspensions was hence incorporated in viral DNA extraction (Additional file 3: Table S2). When performing the optimized protocol, a sufficient amount of pure viral DNA was extracted from 400 to 1000 g of soil (Additional file 3: Table S2, Table 1), which allowed shotgun sequencing devoid of any biasing amplification step prior to library preparation.

**Table 1:** DNA yield, trimmed reads and assembled contigs from the optimized phage extraction routes

Sample	DNA (ng/μl)	Trimmed Reads (Mill)	% of Reads surviving	Contigs (> 5 kb)	Average kb	Nucleotides assembled (Mb)
0.22 μm TFF	2.7	104	94.2	17,698	11.22	196.5
0.22 μm PEG	1.70	60	92.4	7,583	11.15	84.5
0.45 μm TFF	56.6	83	91.0	12,453	11.58	164.0
0.45 μm PEG	2.44	64	92.9	10,493	13.17	121.4

### Metagenomic analysis

By using a spiked phage community as a reporter, it was not possible to provide definite answers regarding an optimal filter pore size (0.22 μm vs. 0.45 μm) or soil phage concentration method (PEG vs. TFF). The four optimized phage DNA extraction routes (0.22 μm + TFF, 0.22 μm + PEG, 0.45 μm + TFF and 0.45 μm + PEG) were hence compared for viral richness, diversity and bacterial DNA contamination levels using metagenomic analysis. Viral DNA was extracted from 1 kg of soil for each extraction route and paired-end shotgun Illumina sequenced with 76 million reads per route. Over 90% of all raw reads survived the initial data pre-processing as trimming and size exclusion and a total of 311 million reads from all four viromes remained. Those reads were assembled into 48,227 contigs (> 5 kb) with an average length of 11.78 kb (Table 1). As a normalization measure and to exclude a potential direct effect of the number of reads to the assembled contigs, a sub-assembly with 60 million reads for each sequenced extraction route was performed (Additional file 4: Table S3). This sub-assembly resulted in less assembled contigs for each virome, indicating an incomplete coverage of the viromic diversity. The abundance of contigs per virome, however, deceased proportionally such that the 0.22 μm + TFF virome still displayed the highest amount of assembled contigs independently from the number of reads used for assembly.

**Fig. 5:**
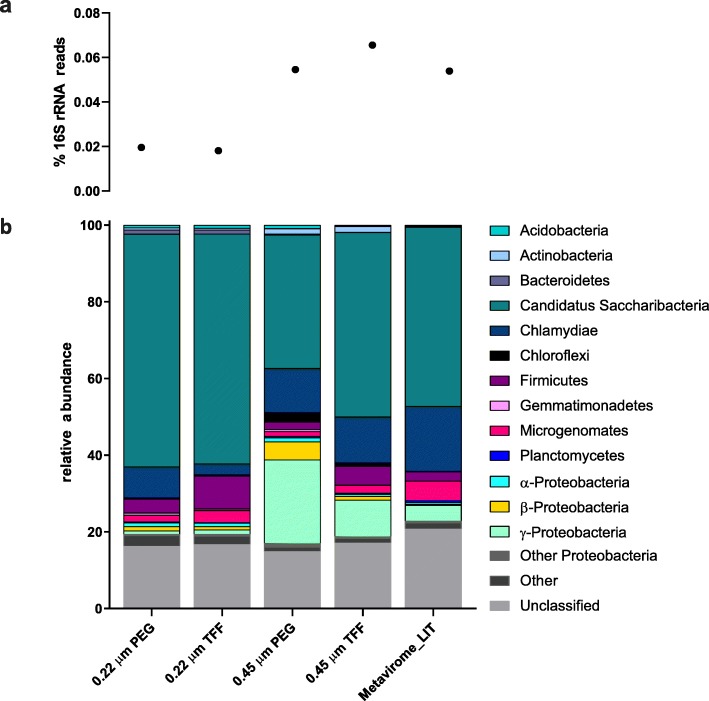
16S rRNA gene contamination. 16S rRNA gene contamination in the five extracted and sequenced viromes. Viromes filtrated with a 0.22 μm filter and extracted with the optimized protocol have contamination levels < 0.02% (threshold for virome purity) [29] (**a**). *Candidatus Saccharibacteria* is the predominant contaminant of all viromes irrespective of the extraction method applied (**b**).

To appraise bacterial contamination levels, the percentage of 16S rRNA reads in each method was assessed based on confirmed 16S rRNA reads after ssu-align, and were taxonomically classified by sequence match against the ribosomal database project (RDP) database [35]. Contamination of 16S rRNA genes in both 0.22 μm filtrated viromes was below 0.2‰ (0.019% in PEG and 0.018% in TFF, respectively). Bacterial DNA contamination in 0.45 μm filtrated viromes was significantly higher and above the suggested threshold for viromes of 0.2‰ (0.054% in PEG and 0.065% in TFF, respectively) [29] (Fig. 5). Similarly, the virome established as proposed in literature (LIT), which is solely filtered through a 0.22 μm PES filter, resulted in equally high contamination levels (0.053%) (Fig. 5). Our proposed optimized protocol resulted, therefore, in a high reduction of external bacterial DNA contamination (> 65%) compared to previously published protocols. Interestingly, each virome, regardless of the phage DNA extraction route, had *Candidatus* Saccharibacteria as the predominant contaminating bacterial phylum (Fig. 5). In the 0.22 μm filtrated viromes, Saccharibacteria composed > 60% of all contaminants, which decreased to 40% in the 0.45 μm filtrated viromes. However, this decrease should not be taken as an absolute value, as 0.45 μm filtrated viromes revealed at least twice as much external contamination (Fig. 5).

After assembly, the 48,227 remaining contigs were manually inspected and classified as viruses, if viral hallmark genes such as terminases or structural proteins were present. Along similar lines, contigs that harboured bacterial genes such as ribosomal sequences were separated from the viral fraction and classified as bacteria. After identification by manual curation, 13,114 contigs were classified as virus and another 13,519 as bacteria, whereas 21,586 remained unclassified due to insufficient annotation (hypothetical proteins or none) (Table 2). Manually classified viral contigs from each virome were then pooled and redundant contigs (clustered at > 99% identity) were removed. This initial clustering analysis resulted in 10,886 (74%) unique and partially complete viral genomes from all four extracted soil viromes. Based on overlapping ends (≥ 10 bp), we could extract 379 novel, circularized phage genomes with sizes between 5.1 and 235 kb (average 58.9 kb) from this non-redundant viral fraction. In addition, 89 putative complete phage genomes (sizes between 5 and 70.9 kb) were identified in contigs left unclassified (Table 3).

**Table 2:** Manually assigned viral, bacterial and unclassified contigs for each sequenced virome

Sample	Viral contigs	Bacterial contigs	Unclassified contigs
0.22 μm TFF	6,345	1549	9801
0.22 μm PEG	2,453	1070	4058
0.45 μm TFF	2,092	7067	3286
0.45 μm PEG	2,224	3833	4441

**Table 3:** Virome contigs after removal of redundant contigs with clustering analysis I and II

	Clustering I	Circularized	Size of circularized genomes	Clustering II
Viral contigs	10,886	379	235–5.1 kb	8835
Bacterial contigs	10,645	–	–	8349
Unclassified contigs	14,180	89	70.9–5 kb	12,520
Total	35,710 (74.0%)	468	235–5 kb	29,704 (61.7%)

**Fig. 6:**
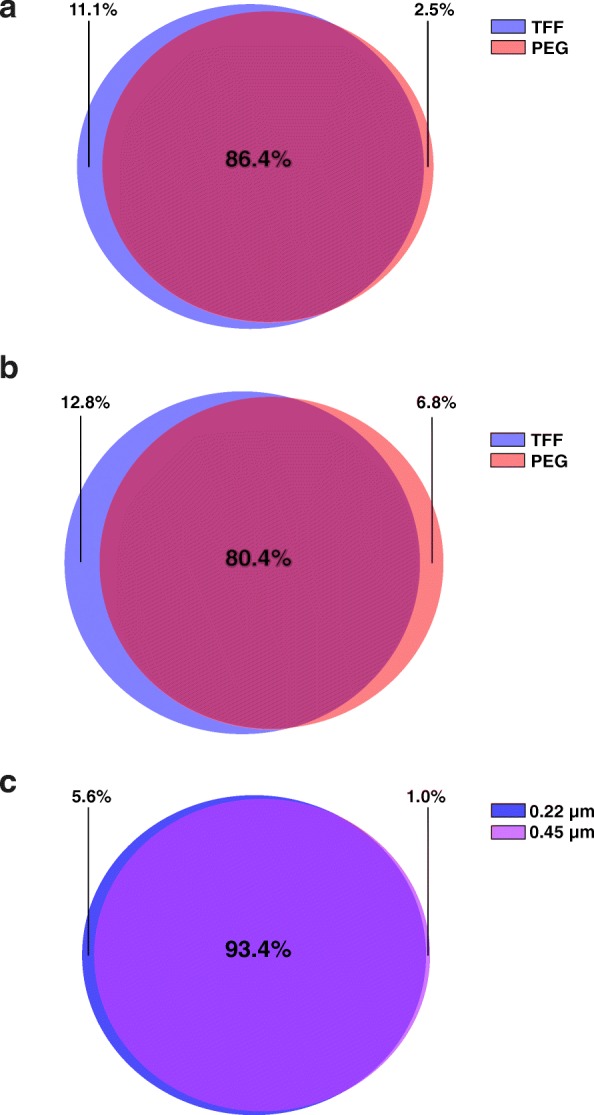
Native soil viral diversity. Recovery of native soil viral diversity in each optimized phage DNA extraction route. The percentage of unique viral contigs recruited within each extraction method is shown. (**a**) Viral diversity in the 0.22 μm filtrated viromes concentrated with either PEG or TFF. (**b**) Viral diversity in the 0.45 μm filtrated viromes concentrated with either PEG or TFF. (**c**) Viral diversity in TFF concentrated viromes, filtrated through either 0.22 μm or 0.45 μm PES filter.

Prior to the assessment of viral diversity, all redundant viral contigs were removed with clustering 1 and 2 information, leaving 29,704 (61.7%) contigs classified as either viral, bacterial or of unknown origin (Table 3). A subset of 20 million reads from each sequenced virome was then separately mapped against this manually curated and trimmed viral community (8835 unique viral contigs) to estimate viral recruitment in each extraction method. In the 0.22 μm filtrated viromes, 97.5% of all recovered viruses were present in the TFF route (967 unique viral contigs > 5 kb), whereas 88.9% were recovered by PEG concentration (219 unique viral contigs > 5 kb) (Fig. 6a). Similarly, by comparing TFF versus PEG concentration in the 0.45 μm filtrated viromes, it is evident that TFF performed better by recovering a higher percentage of unique soil viruses (Fig. 6b).

**Fig. 7:**
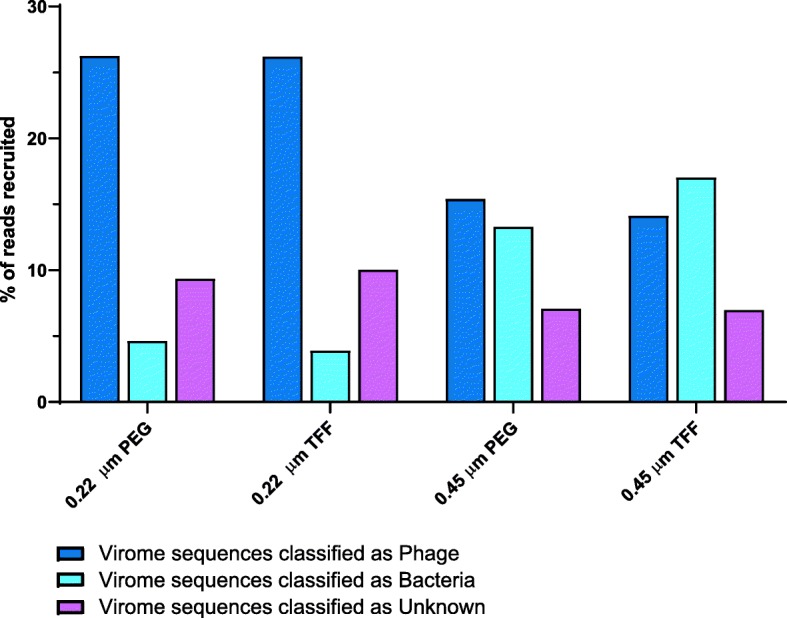
Virome read association. Percentage of reads recruited to the manually classified viral, bacterial or unknown contigs for each virome.

Viromes filtrated with a 0.45 μm filter (0.06% 16S rRNA reads) opposed to a 0.22 μm filter (0.018% 16S rRNA reads) harboured 70% more bacterial DNA contamination (Fig. 5), and failed to recover unique soil viruses. Indeed, the 0.45 μm filtrated viromes recovered 94.4% of all viral contigs and therefore consisted of less unique soil viruses compared to a 0.22 μm filtrated virome (99% recovery) (Fig. 6c). In addition to viral diversity, variations in the percentage of reads that matched to viral, bacterial or unknown contigs were observed. The percentage of reads that matched with bacterial contigs extracted from the 0.22 μm and 0.45 μm filtrated optimized protocols were 4.2% and 15.1%, respectively, confirming the reduced bacterial contamination in the 0.22 μm filtrated viromes. In addition, the 0.22 μm filtrated viromes displayed the highest sequence affiliation to viral contigs, which recruited more than 25% of all reads. Recruitment of viral reads decreased to less than 15% in the 0.45 μm extracted viromes (Fig. 7). Notably, the recruitment rates for viral contigs in the unclassified fraction, which reflect contigs with no bacterial annotation, was also considerably higher in the 0.22 μm filtrated samples.

## Discussion

We here report the optimization of protocols for extraction of bacteriophage dsDNA from soil preceding metagenomic analysis. As anticipated, the elution of virus particles from soil samples has crystallized to be the major bottleneck in the present study [1, 2], due to the high absorption of bacteriophages to soil particles (> 90%) [27]. Indeed, all suggested elution buffers in the literature [1, 5, 18, 23, 24, 28] were either found to perform insufficiently in the recovery of bacteriophages from agricultural soil (< 5% of spiked phages) or resulted in major technical limitations due to complete inhibition of downstream filtration procedures. We designed an optimal elution buffer PPBS, consisting of ionic salt compounds supplemented with 2% BSA that disrupts phage soil particle interactions through competing for viral binding sites. This finding is consistent with Lasobras et al. [24], who reported that an optimal elution of virus particles from soil requires either a proteinaceous material that competes for viral binding sites or chaotropic agents which alter the favourability of absorption. The beneficial action observed with PPBS buffer was not enhanced when substituting BSA with beef extract, which supports the favourable effect of BSA in viral elution. In summary, the optimized elution protocol described here results in a substantial recovery of spiked bacteriophages, whereas no technical, difficult or harsh method was applied to maximise viral retrieval. As a major advantage, this gentle optimized elution protocol potentially allows the isolation of infective viral particles through omitting techniques that could result in tail breakages or defective particles.

For shotgun sequencing or functional metagenomics, a maximum reduction of bacterial DNA contamination is crucial to allow data analysis. Filtration of unknown viral suspensions to remove bacterial contamination is, therefore, an extensively discussed topic in previous studies. Published protocols in literature suggest either a 0.22 μm, 0.45 μm or 0.8 μm filtration of extracted viromes to decrease bacterial contamination below a threshold and simultaneously not impair viral yield or diversity. As shown by shotgun sequencing of 0.22 μm and 0.45 μm filtrated viromes, a 0.22 μm PES filter removed more bacterial DNA contamination compared to a 0.45 μm PES filter, while simultaneously not compromising soil viral diversity and recovery. Indeed, less viral diversity was observed in viromes filtrated through a 0.45 μm pore-size, which is most likely due to the increased bacterial DNA contamination and thus, the impaired assembly of viral contigs (Fig. 6). This finding is supported by our recruitment analysis, which revealed similar amounts of reads recruited to the manually classified viral or bacterial fraction (14.7% and 15% respectively) in 0.45 μm viromes. The vast majority of reads recruited in the 0.22 μm viromes, however, matched to viral contigs and < 5% to bacteria (Fig. 7). Using the proposed optimized protocol for elution and filtration of soil viruses, the 16S rRNA gene contamination could be reduced to a level below the recommended threshold of 0.2‰ [29]. This finding is consistent with Castro et al. [28], who observed a considerably lower host DNA contamination when relying on both centrifugation (e.g. thrice 5000×*g*) and filtration techniques. Interestingly, the predominant bacterial contaminant in all sequenced viromes was found to be *Ca*. Saccharibacteria*.* They are exceptionally small Gram-positive cocci (200–300 nm) that are able to pass through a 0.22 μm filter due to their size [36]. These bacteria are thus being concentrated along with bacteriophages in all protocols, and their DNA extracted alongside. Unfortunately, *Ca*. Saccharibacteria are not only found in soil, but in many other environmental samples such as sludge and activated sludge from wastewater treatment plants, human saliva and the gut microbiome [36]. Any virome extracted from those samples must, therefore, be either manually curated to remove bacterial reads, or handled with great care to reach valid conclusions.

In order to concentrate bacteriophages from large volumes, the most commonly used approaches such as TFF and PEG precipitation were compared for efficiency here. Independently from the initial filtration technique applied, TFF performed better in recovering and concentrating soil phages and, therefore, revealed a greater soil viral diversity as assessed using sequencing analysis. After concentration, a purification of soil viral suspensions using CsCl ultracentrifugation was implemented in the optimized protocol before viral DNA extraction to remove inhibitors and allow ultrafiltration concentration to a final volume below 300 μl. As resolved here, CsCl ultracentrifugation needs to be applied for purification purposes prior to DNA extraction when working with large amounts of soil, but could be omitted when aiming for the isolation of infective viral particles or the extraction of phages from small amounts of soil [21]. Furthermore, the densities of the CsCl gradient can be adapted according to Trubl et al. to possibly capture ssDNA viruses [21].

After manual classification of 48,227 assembled contigs (> 5kb) from the four extracted viromes, we confirmed 10,886 contigs as unique partial viral genomes and another 14,180 as putative viral contigs without bacterial hallmark genes. This finding roughly doubles the currently 27,502 published viral contigs from soil [37], and highlights the still very fragmented nature of available datasets from soil ecosystems. Out of our manually identified viral fraction, 379 novel and presumably complete phage genomes, which average size is consistent with presently isolated dsDNA viruses [38], were extracted. In addition, we obtained another 89 possibly closed and novel putative phage genomes in length up to 70.9 kb from the contigs that were classified as unknown. These numbers are even more noticable when compared to the recently 999 extracted complete viral genomes (> 100 bp overlapping ends) from over 125,000 contigs that derived from 3042 geographically diverse environmental samples [38]. In summary, our newly developed protocol for the extraction of soil bacteriophages has proven to produce robust results not only in a culture dependent analysis through spiked bacteriophages but also through sequencing viromes and promises exciting insights into the immense viral diversity of the previously largely inaccessible soil virome.

## Conclusion

To our knowledge, this is the first optimized bacteriophage extraction protocol for agricultural soil, which has proven robustness through viromic analysis and could be extrapolated for infective phage isolation due to gentle elution techniques. We have shown a dramatically enhanced extraction of the soil phage community by protocol optimization and present soil viromes harbouring 468 novel, possibly complete soil bacteriophages in over 25,000 non-bacterial contigs. Our huge data set of manually curated soil viral contigs provides insights into the yet largely undescribed soil viral sequence space and is the most comprehensive study to date. Our optimized protocol could be valuable for any viral extraction from solid matrixes, given the development of an adequate phage elution buffer for the respective sample. Our guidelines for the elution process and buffer design will facilitate such proposals in the future.

## Methods

### Soil samples

Agricultural soil samples were collected from a long-term soil experimental field (ZOFE, Zurich Organic Fertilization Experiment), located in a rural area surrounding Zurich (Agroscope, Reckenholz, Switzerland). Soil consisted of 56% sand, 28% silt and 14% clay (in mass %: 0.6 soil organic carbon, 1.1 soil humus, pH ~ 5.7). Soil samples for spiking and optimization of extraction routes were taken in September 2017 (replica I-V, unfertilized control samples), while those for sequencing metagenomics were taken in October 2018 (replica I–V, farmyard manure fertilized samples). For each replica plot, the first 10 cm of superficial soil was sampled randomly six times with equal distances apart, and the upper rhizosphere (2–3 cm) removed. Samples were passed through a 2 mm pore size sieve. Replica plots were combined in batches of 400 g or 1000 g, and stored immediately at − 80 °C. Prior usage, the soil was defrosted at 4 °C for 4 h.

### Design of viral mock-community

For accurate phage quantification, three strictly lytic dsDNA phages were propagated, and each spiked at a concentration of 1 × 10^6^ pfu/g soil: A511 (Myovirus), 2638AΔLCR (Siphovirus) and T7 (Podovirus). Phages were chosen due to their absence in soil and as representatives of the three families belonging to the dsDNA most-abundant bacteriophage order: *Caudovirales.* Enterobacteria phage T7 and *Listeria* phage A511 were obtained from our in-house stock. *Staphylococcus* phage 2638AΔLCR is a modified version of phage 2638A lacking the lysogenic control region (LCR) (Samuel Kilcher, unpublished), and was chosen to assess the rate of recovery of phages infecting this bacterial genus.

### 16S rRNA gene qPCR analysis

The presence of contaminating bacterial DNA was assessed throughout each extraction route (Additional file 1: Figure S1) using Taqman 16S rRNA qPCR. For this, a 16S rRNA gene fragment (934 bp) was cloned into a pGEM-T-Easy-Vector (3015 bp), and the correct insertion was verified using restriction enzyme digestion. For standard preparation, the plasmid containing the insert was linearized and purified with Gene-Elute PCR Clean-Up Kit (Sigma). DNA concentration was measured with Qubit (ThermoFisher), and copy numbers were calculated. Taqman qPCR was carried out using the SensiFAST Probe no-ROX kit (Bioline), whereas primer and probes were placed in conserved regions of the 16S rRNA gene (amplicon size: 105 bp, Additional file 2: Table S1). All qPCR assays were performed on Rotorgene 600 (BioLabo, Corbett Research) with following conditions: 5 min at 95 °C for polymerase activation, followed by 40 cycles at 95 °C for 10 s and 60 °C for 20 s.

### Plaque assay

Spiked bacteriophage recovery was quantified at each of the optimization steps (Additional file 1: Figure S1), using plaque assays. Phage A511, 2638AΔLCR and T7 were titrated on host strains *Listeria ivanovii* WSLC 3009, *Staphylococcus aureus* 2638A and *Escherichia coli* DSM496, respectively. All plaque assays were carried out using LC agar as top agar (10 g/L casein pepton, 5 g/L yeast extract, 128 mM NaCl, 55.5 mM glucose, 2 mM MgSO_4_, 10 mM CaCl_2_, 0.4% agar). As bottom agar, brain heart infusion agar (18.5 g/L, 2% agar) was used for *L*. *ivanovii* 3009 and *S*. *aureus* 2638A, and Luria-Bertani agar (per litre, 10 g casein peptone, 5 g yeast extract, 7 g NaCl, 2% agar, pH 7.2) for *E*. *coli* DSM496. Phages (10 μl, serial diluted) were mixed with hosts in molten soft agar (47 °C), and plates were incubated for 16 h prior quantification at 37 °C for *E*. *coli* DSM496 and *S*. *aureus* 2638A, and at 30 °C for *L*. *ivanovii* 3009. The absence of spiked phages (100 μl, undiluted) in each soil sample was confirmed by plaque assay from eluted soil on all host strains.

### Elution of bacteriophages from soil

The elution optimization strategy is summarized in Fig. 1. Soil samples (400 g) were spiked for each phage with 1 × 10^6^ pfu g^−1^ soil. The spiked sample was suspended 1:1 (w/v) in the respective elution buffer and manually shaken for 10 min by repetitive inversion. Elution buffers previously proposed in literature, such as SM buffer [11, 23] (200 mM NaCl, 10 mM MgSO_4_, 50 mM tris and 0.01% gelatin, pH 7.4), 10% beef extract buffer (10% beef extract in ddH_2_O) [5, 24], PBS supplemented with beef extract (PBS, 2.5% beef extract, pH 8.5) and AKC [1] (1% potassium citrate, 10% PBS, 150 mM MgSO_4_, 5 mM EDTA) were tested for efficiency*.* Those elution buffers were additionally altered in constitution and assessed for efficacy with the following modifications: SM buffer was supplemented with 0.01% tween or reduced in magnesium (5 mM MgSO_4_), and elution buffers PPBS (2% BSA, 10% PBS, 1% potassium citrate, 150 mM MgSO_4_) and BPBS (2% beef extract, 10% PBS, 1% potassium citrate, 150 mM MgSO_4_) were created. After the gentle elution, soil samples were left in suspension overnight at 4 °C. The next day, suspended soil samples were either directly subjected to bacterial DNA elimination (Fig. 1, route 1–4), or elsewise, remaining soil pellets were resuspended twice more (Fig. 1, route 5–6). For this, the eluted overnight soil sample was centrifuged 10,000×*g* [6, 11], 10 min at 4 °C and the first supernatant kept aside. Consecutively, the same soil pellet was again suspended in equal parts of the buffer, put on a shaker for 30 min at 300 rpm, 4 °C and centrifuged as described above. This was repeated a third time to maximise bacteriophage recovery [6, 12]. PFU for each spiked phage was assessed in all supernatants and the three finally united.

### Removal of bacterial contamination

In order to reduce contaminating bacteria and sediments, the single (Fig. 1, route 1–4) or united (Fig. 1, route 5–6) supernatants were centrifuged three rounds at 5000×*g* [28], for 10 min at 4 °C. At each individual round, the supernatant was recovered into a new, sterile centrifugation tube and the pellet discarded. To remove larger floating particles that were not parted by centrifugation, soil supernatants were pre-filtrated using a 16 μm cellulose filter and sterile glassware. The filtrate was eventually passed through either a 0.45 μm or 0.22 μm PES filter. Bacterial contamination, as well as recovered bacteriophages, were determined using 16S rRNA gene qPCR and plaque assays, respectively. Besides centrifugation and filtration, the efficiency of chloroform treatment to remove bacterial contamination was assessed. To evaluate potential benefits of chloroform and to concurrently allow downstream concentration of viral particles, a final concentration of 0.8% chloroform was applied (Fig. 1, route 1–2).

### Tangential flow filtration

For concentrating soil viral particles, a TFF approach was tested (Fig. 1, route II–III). Briefly, viral suspensions were concentrated using a 100 kDa cut-off PES membrane (Millipore) and the retentate containing the bacteriophages (> 100 kDa) was continuously cycled to maximally reduce its volume. The presence of spiked bacteriophages was quantified in the retentate and their absence confirmed in the permeate. TFF concentrated soil viral suspensions were either purified with CsCl ultracentrifugation, or directly subjected to DNA extraction.

### Polyethylene glycol precipitation

Concentration of soil viral particles using PEG precipitation was performed as follows (Fig. 1, route I). Soil suspensions were mixed thoroughly 2:1 with a 3× precipitant solution (30% PEG 6000 and 3 M NaCl in autoclaved ddH_2_O), and put in ice-water at 4 °C overnight. Next, the suspensions were centrifuged at 16,000×*g* for 1 h at 4 °C and the pellet resuspended in 7 ml of SM buffer. To free the concentrated viral particles of PEG, samples were dialyzed in 4 L of SM buffer overnight at room temperature (RT) using a 50 kDa membrane (Biotech CE tubing).

### Caesium chloride gradient purification

Concentrated viral particles were purified using ultracentrifugation in a four-layered CsCl gradient (Fig. 1, route I–II). Methods were adapted from literature [23, 28]. Briefly, 10 ml of a concentrated sample was adjusted to a density of 1.15 g ml^−1^ and loaded on top of a 6 ml step gradient containing 2 ml of 1.35, 1.5 and 1.7 g ml^−1^ CsCl, respectively. Gradients were centrifuged at 82,000×*g* for 2 h at 10 °C. Bacteriophages in the density fractions between 1.35 and 1.5 were harvested (position visible through a light blue phage band). The collected samples with a final volume of 2–3 ml per gradient were dialyzed at 4 °C, as described above.

### Ultrafiltration concentration

The TFF retentate and dialyzed CsCl fractions were further concentrated using Amicon ultra centrifugal filters with 100 kDa cut-off (Millipore) (Fig. 1, all routes). Prior to centrifugation, filters were coated with PBS + 2% BSA in order to prevent viral absorption [34]. The concentrated sample was recovered into a sterile Eppendorf, and the centrifugal filter washed twice with 100 μl of ddH_2_O. Bacteriophage recovery in the concentrate and bacteriophage absence in the filtrate was confirmed using plaque assay. The total volume was eventually brought up to 450 μl for DNA extraction.

### Viral DNA extraction

The optimization strategy for viral DNA extraction is summarized in Fig. 1 (route A–E). Phenol chloroform viral DNA extraction was carried out as described elsewhere [23] with some optimizations. Ultrafiltrated concentrated samples were supplemented with 50 μl of 10× DNase I Buffer (ThermoFisher) and treated with 10 U of DNase I (ThermoFisher) for 2 h at 37 °C. The enzyme was inhibited using 50 mM EDTA at 65 °C for 10 min, and the volume brought up to 600 μl. From here, viral DNA was either extracted using modified phenol/chloroform extraction routes (Fig. 1, route A–D) or QIAamp Viral RNA Mini Kit [30] (QIAGEN) according to manufacturer’s instructions (Fig. 1, route E).

For phenol/chloroform viral DNA extraction, the volume was split into two Eppendorf. Eppendorf 1 (route A–B) was treated, following recommendations in Thurber et al. [23] as follows: 0.1 volumes of 2 M Tris HCL/0.2 M EDTA, 1 volume of formamide and 1 μl glycogen at 20 mg/ml were added to each sample. Straight after incubation at RT for 30 min, DNA was spun down by adding two volumes of 99.9% ethanol and centrifuged at 14,000×*g* for 20 min at 4 °C. The pellet was washed twice using 70% ice-cold ethanol and resuspended overnight in 300 μl of TE buffer (10 mM Tris, 0.5 M EDTA, pH 8) at 4 °C. The remaining suspension was again split into two equal volumes. Eppendorf 2, which was not treated with formamide, was equally split (route C–D). All four Eppendorf tubes were topped up to 567 μl using sterile ddH_2_O and the DNA was extracted as follows: 30 μl of 10 % sodium dodecyl sulfate (SDS) and 3 μl of 20 mg/ml Proteinase K were added, mixed and incubated for 1 h at 55 °C. Subsequently, in one Eppendorf originating from the formamide treatment (route A), and one without formamide treatment (route C), 80 μl of CTAB/NaCl solution was added and incubated for 10 min at 65 °C. Finally, all samples were identically treated in accordance with established protocols [23]: equal volumes of chloroform were added, and samples were centrifuged for 5 min at 8000×*g* at RT. The supernatant of each route was transferred to a separate tube and equal volumes of first phenol/chloroform/isoamyl alcohol (25:24:1) and subsequently chloroform were added, and centrifuged at the same conditions as above. After the second chloroform treatment, the supernatant was recovered, and 0.7 volumes of isopropanol were supplemented to precipitate the DNA overnight at 4 °C. The next day, all samples were centrifuged for 15 min at 13,000×*g*, 4 °C, and the pellet was washed with 500 μl of 70% ice-cold ethanol. The ethanol was then removed, the pellet air-dried and resuspended in 50 μl of ddH_2_O overnight. This DNA extraction optimization protocol was applied to larger volumes if the sample was not ultracentrifuged but directly originated from TFF concentration.

### Library preparation, Illumina sequencing and annotation

The four most optimal extraction routes, e.g. 0.22 μm + TFF, 0.22 μm + PEG, 0.45 μm + TFF and 0.45 μm + PEG, were selected based on spiked phage recovery and bacterial depletion. Those extraction protocols were then used to extract viral DNA originating from 1 kg of freshly agricultural soil (ZOFE, see above) and shotgun sequenced. For this, soil samples were suspended in PPBS, filtrated, concentrated and purified with CsCl ultracentrifugation. Viral DNA was obtained using the optimized DNA extraction protocol, including CTAB but neglecting formamide. Libraries were prepared with 25 ng unamplified viral DNA of each respective route using NebNext Ultra II DNA Library Prep for Illumina and following the manufacturer’s instructions (ten rounds of PCR amplification). Library pooling and normalization was based on the concentration of the final libraries as determined with Tapestation (Agilent 4200). Tagged libraries were sequenced with 76 million paired-end reads (150 bp/read) using NextSeq 500 sequencing. Raw reads were trimmed with Trimmomatic in default settings and unshuffled trimmed reads paired into a single file using shuffleSequences [39, 40]. Shuffled sequences from each individual virome were assembled with IDBA-UD [41] and contigs larger than 5 kb were extracted for further analysis. Open reading frames (ORFs) on assembled contigs (> 5 kb) were predicted using prodigal [42] and annotated using DIAMOND [43] against the NCBI NR, and using HMMscan [44] against the Pfam [45], COGs [46] and TIGRfams [47] databases.

### 16S rRNA gene contamination

In order to assess 16S rRNA gene contamination in the four sequenced viromes, reads from each sample were trimmed to a minimum length of 50 bp, and a random subset of 20 million trimmed reads were kept for further analysis. Potential 16S rRNA DNA reads were retrieved by USEARCH6 [48] against the RDP database [49], previously clustered at 90% identity. The percentage of 16S rRNA gene reads in each method was then calculated based on hits that were confirmed by ssu-align [50]. Affirmed 16S rRNA gene reads were taxonomically classified using the RDP database and classifications with a sequence match (S_ab score) higher than 0.8 were kept. In order to compare the efficiency of bacterial DNA removal with the here optimized protocols, 16S rRNA gene reads originating from a virome extracted from the same soil but using a former standardised protocol published in the literature (LIT) [23, 28] was also processed as described above.

### Classification of contigs and cluster analysis for complete viral genomes

In a first step, annotated contigs (> 5 kb) from the four sequenced viromes were manually inspected and classified. Contigs were assigned as viral if phage structural genes such as terminase, portal, capsid or tail proteins were present, or if the majority of taxonomical hits belonged to virus. Elsewise, contigs with ribosomal proteins, cell division proteins or other bacteria hallmark proteins were classified as bacteria. Contigs with no evident gene indicators or contigs with proteins of none or hypothetical functions were left as unclassified [29]. Manually assigned viral contigs were then pooled together and redundant viral sequences removed. For this, all viral contigs were globally aligned [51] and clustered at > 99% identity, whereas only the largest representative contig of each cluster was kept. Phage genomes were then assessed for completeness by searching for overlapping nucleotide sequences (> 10 bp) at the 3′ and 5′ region.

### Viral recruitment comparison in phage extraction routes

Viral diversity in each extraction route was compared by mapping a subset of 20 million reads from all four viromes to the total extracted soil viral community. This viral community originated from the manually curated viral contigs that survived the first and a second cluster round. For the second cluster analysis, remaining viral contigs were anew clustered, if more than 30% of a smaller contig was present at > 99% identity in a larger contig (local alignment) [51]. Reads of each virome were then mapped against all viral contigs that were cleared for redundant sequences, and phage abundance and diversity from each optimized method was analysed. To provide a normalized measure, the number of hits to each phage contig was divided by the length of the contig (in kb) and by the size of the virome (size of the database in Gb). This measure is abbreviated as RPKG (reads per Kb per Gb) and helps to compare recruitments by differently sized contigs versus several metagenomes. A phage was considered present in a given virome, if the contig was covered by at least by 1 RPKG at 98% identity.

### Virome reads associated with bacterial, viral or unclassified contigs

A subset of 20 million reads of each extraction route was mapped to the manually classified viral, bacterial or unknown contigs. For this, all viral sequences obtained from the extracted viromes were concatenated to one super-viral DNA contig. All bacteria or unknown sequences were likewise combined. This concatenation prevents multiple mapping of a single read to a given viral sequence if represented several times in the assembled viromes. The total percentage of reads recruited to either the phage, bacterial or unclassified concatenated super contig was then assessed at 98% identity.

## Supplementary information

**Additional file 1: Figure S1.** Optimization Strategy (PDF). Optimization strategy of phage extractions protocols form soil samples prior viromic analysis. Different phage elution, filtration, concentration and DNA extraction procedures were tested to maximise viral yield and deplete bacterial DNA contaminants. *16S rRNA qPCR to determine external contaminants, ^Ɨ^ plaque assay to assess spiked bacteriophage recovery.

**Additional file 2: Table S1.** Primer and probes for 16S rRNA qPCR (PDF). Primer and probes designed for TaqMan 16S rRNA gene qPCR.

**Additional file 3: Table S2**. DNA yield and external contamination with phage DNA extraction methods (PDF). DNA yield and bacterial DNA contamination of phage DNA extraction routes from soil samples.

**Additional file 4: Table S3.** Assembly with 60 million reads (PDF). Normalized assembly of 60 million reads for the extracted soil viromes.

## Data Availability

Metagenomic datasets have been submitted to NCBI SRA and are available under BioProject accession number PRJNA544697 (ZOFE18-TFF_0.22 [SAMN11866222], ZOFE18-TFF_0.45 [SAMN11866225], ZOFE18-PEG_0.22 [SAMN11866227], ZOFE18-PEG_0.45 [SAMN11866228] and ZOFE16-VIR-MAN [SAMN11866229]).

## **Abbreviations**

AKC: Amended potassium citrate
BPBS: Beef extract supplemented PBS
BSA: Bovine serum albumin
CsCl: Caesium chloride
CTAB: Cetyltrimethylammonium bromide
EDTA: Ethylenediaminetetraacetic acid
LIT: Literature suggested buffer or protocol
O/N: Overnight
PBS: Phosphate-buffered saline
PEG: Polyethylene glycol
PES: Polyethersulfone
Phages: Bacteriophages
PPBS: Protein supplemented PBS
RDP: Ribosomal database project
RPKG: Reads per Kb per Gb
RT: Room temperature
SDS: Sodium dodecyl sulfate
SM buffer: Saline magnesium buffer
TFF: Tangential flow filtration
w/v: Weight to volume
