# Supplementary material for: Uncovering a hidden diversity: optimized protocols for the extraction of dsDNA bacteriophages from soil
Source: Microbiome. 2020 Feb 11;8:17. doi: 10.1186/s40168-020-0795-2 (PMC7014677; doi:10.1186/s40168-020-0795-2)
Supplement: Supplementary file 1 — Additional file 1: Figure S1. Optimization Strategy (PDF). Optimization strategy of phage extractions protocols form soil samples prior viromic analysis. Different phage elution, filtration, concentration and DNA extraction procedures were tested to maximise viral yield and deplete bacterial DNA contaminants. *16S rRNA qPCR to determine external contaminants, Ɨ plaque assay to assess spiked bacteriophage recovery. [file 40168_2020_795_MOESM1_ESM.pdf]

Additional file 1: Figure S1

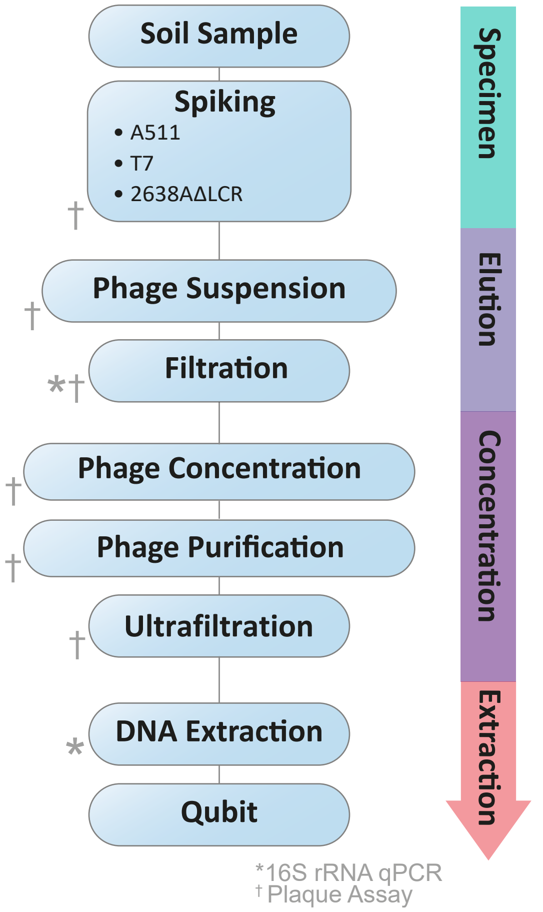

Figure S1. Optimization strategy of phage extractions protocols form soil samples prior viromic analysis. Different phage elution, filtration, concentration and DNA extraction procedures were tested to maximise viral yield and deplete bacterial DNA contaminants. *16S rRNA qPCR to determine external contaminants, ƚ plaque assay to assess spiked bacteriophage recovery.
