# Supplementary material for: Uncovering a hidden diversity: optimized protocols for the extraction of dsDNA bacteriophages from soil
Source: Microbiome. 2020 Feb 11;8:17. doi: 10.1186/s40168-020-0795-2 (PMC7014677; doi:10.1186/s40168-020-0795-2)
Supplement: Supplementary file 2 — Additional file 2: Table S1. Primer and probes for 16S rRNA qPCR (PDF). Primer and probes designed for TaqMan 16S rRNA gene qPCR. [file 40168_2020_795_MOESM2_ESM.docx]

**Additional file 2: Table S1**

Table S1. Primer and probes designed for TaqMan 16S rRNA gene qPCR.

| **Oligos** | **Sequence (5'-3')** |
| --- | --- |
| Primer_fw | GCGGTGAAATGCGTAGAGAT |
| Primer_rv | TCTAATCCTGTTTGCTCCCCA |
| Probe | FAM-GCGAAGGCGGCCCCCTGGAC-BHQ1 |
