# Supplementary material for: Uncovering a hidden diversity: optimized protocols for the extraction of dsDNA bacteriophages from soil
Source: Microbiome. 2020 Feb 11;8:17. doi: 10.1186/s40168-020-0795-2 (PMC7014677; doi:10.1186/s40168-020-0795-2)
Supplement: Supplementary file 3 — Additional file 3: Table S2. DNA yield and external contamination with phage DNA extraction methods (PDF). DNA yield and bacterial DNA contamination of phage DNA extraction routes from soil samples. [file 40168_2020_795_MOESM3_ESM.docx]

**Additional file 3: Table S2**

Table S2. DNA yield and bacterial DNA contamination of phage DNA extraction routes from

soil samples.

| **CsCl Purified Sample** | **16S rRNA (copies/μL)** | **Inhibition** | **DNA in 400 g of soil (ng/μL)** |
| --- | --- | --- | --- |
| No Formamide, no CTAB | 2.22E+03 | - | 12.4 |
| No Formamide, CTAB | 3.73E+02 | - | 24.4 |
| Formamide, no CTAB | 1.81E+03 | - | 10.2 |
| Formamide, CTAB | 1.56E+03 | - | 6.2 |
