# Supplementary material for: Uncovering a hidden diversity: optimized protocols for the extraction of dsDNA bacteriophages from soil
Source: Microbiome. 2020 Feb 11;8:17. doi: 10.1186/s40168-020-0795-2 (PMC7014677; doi:10.1186/s40168-020-0795-2)
Supplement: Supplementary file 4 — Additional file 4: Table S3. Assembly with 60 million reads (PDF). Normalized assembly of 60 million reads for the extracted soil viromes. [file 40168_2020_795_MOESM4_ESM.docx]

**Additional file 4: Table S3**

Table S3. Normalized assembly of 60 million reads for extracted soil viromes.

| **Sample** | **Trimmed Reads (Mill)** | **Contigs (> 5 kb)** | **Nucleotides Assembled (Mb)** |
| --- | --- | --- | --- |
| 0.22 μm TFF | 60 | 11,215 | 125.1 |
| 0.22 μm PEG | 60 | 7,577 | 84.5 |
| 0.45 μm TFF | 60 | 9,517 | 110.5 |
| 0.45 μm PEG | 60 | 9,288 | 117.5 |
